# Supplementary material for: Rab32/38-Dependent and -Independent Transport of Tyrosinase to Melanosomes in B16-F1 Melanoma Cells
Source: Int J Mol Sci. 2022 Nov 16;23(22):14144. doi: 10.3390/ijms232214144 (PMC9695596; doi:10.3390/ijms232214144)
Supplement: Supplementary file 1 [file ijms-23-14144-s001.zip › ijms-2001463-supplementary.pdf]

Supplemental Information

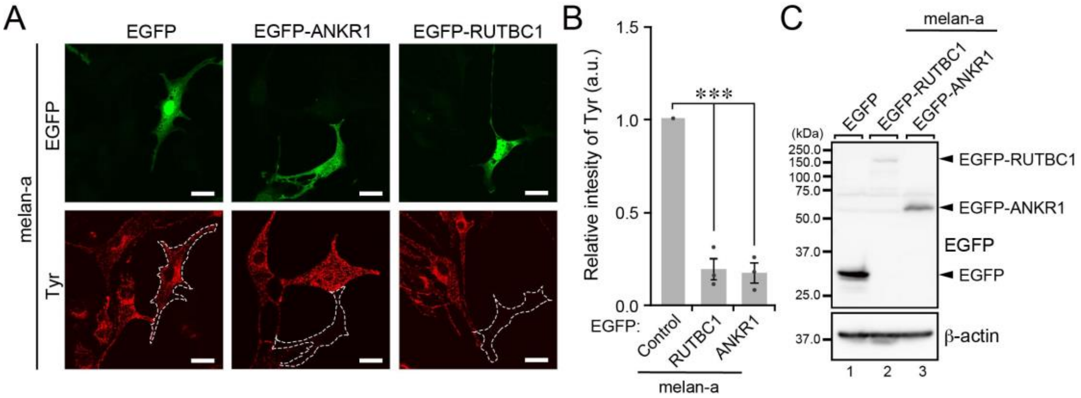

Figure S1. Overexpression of EGFP-tagged RUTBC1 and Varp-ANKR1 in melan-a cells resulted in a dramatic decrease in Tyr signals. (**A**) Representative Tyr images of melan-a cells expressing either EGFP alone, EGFP-RUTBC1, or EGFP-ANKR1. The EGFP-expressing cells are outlined with broken white lines. Scale bars, 20 μm. (**B**) Quantification of the Tyr signals in melan-a cells shown in (**A**). The graph shows the means and SEM of the data obtained in three independent experiments (n > 5 cells in each experiment). ***, $p < 0.001$; NS, not significant (Tukey's test). (**C**) Expression of EGFP-tagged proteins in melan-a cells. Lysates of the cells indicated were analyzed by immunoblotting with antibodies against GFP (top) and β-actin (bottom).

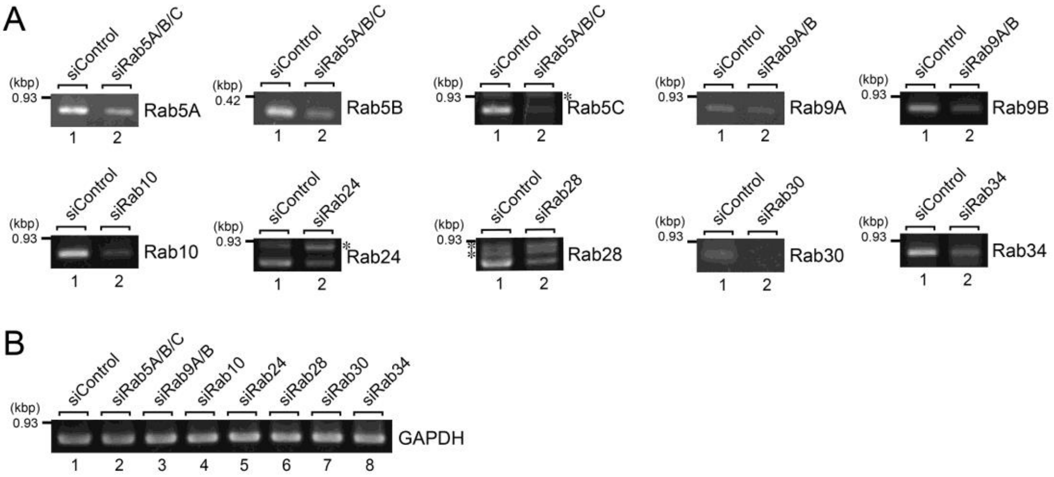

Figure S2. KD efficiency of the candidate Rabs whose KD resulted in a decrease in Tyr signals in *Rab32/38*-KO B16-F1 cells. (**A**) RNAi-mediated KD of the candidate Rabs in *Rab32/38*-KO cells (see Figure 6A) as revealed by an RT-PCR analysis. The authenticity of each band was confirmed by direct sequencing of the PCR products. The asterisks indicate nonspecific bands. (**B**) GAPDH expression is shown for reference to ensure that equivalent amounts of first-strand cDNA were used for the analysis shown in (**A**). The size of the molecular mass markers (kbp, kilobase pair) is shown on the left side of each panel.

Table S1. List of materials used in this study.

Oligonucleotides	Sequence (5'-to-3' direction)	Source
siRNAs		
mouse *Rab32* siRNA	CAACATGACTCGAGTATAC	Nippon Gene (Toyama, Japan)
mouse *Rab1A*–*Rab43* siRNA#1 collection	See Table S1 in *EMBO Rep.* (2013) 14, 450-457	*EMBO Rep.* (2013) 14, 450-457
mouse *Rab10* siRNA#2	CAAGAGAGTTGTACCGAAA	Nippon Gene (Toyama, Japan)
mouse *Rab24* siRNA#2	CTATGCCGATAATATCAAA	Nippon Gene (Toyama, Japan)
mouse *Rab34* siRNA#2	TGGAACGATTTGAAGTCTT	Nippon Gene (Toyama, Japan)
sgRNA primers		
Tyr-sgRNA-fw	CACCGAGTCCCTGCGGCCAGCTTTC	Eurofins Genomics (Tokyo, Japan)
Tyr-sgRNA-rv	AAACGAAAGCTGGCCGCAGGGACTC	Eurofins Genomics (Tokyo, Japan)
Hps4-sgRNA-fw	CACCGTTACCTTGGAACCGTCATAC	Eurofins Genomics (Tokyo, Japan)
Hps4-sgRNA-rv	AAACGTATGACGGTTCCAAGGTAAC	Eurofins Genomics (Tokyo, Japan)
Rab27A-sgRNA-fw	CACCGAACCCAGATATAGTGCTGTG	Eurofins Genomics (Tokyo, Japan)
Rab27A-sgRNA-rv	AAACCACAGCACTATATCTGGGTTC	Eurofins Genomics (Tokyo, Japan)
Ra32-sgRNA-fw	CACCGAATGGTGGCGCGGTAGTGCT	Eurofins Genomics (Tokyo, Japan)
Rab32-sgRNA-rv	AAACAGCACTACCGCGCCACCATTC	Eurofins Genomics (Tokyo, Japan)
Ra38-sgRNA-fw	CACCGTGGTGTGGACTTCGCGCTGA	Eurofins Genomics (Tokyo, Japan)
Rab38-sgRNA-rv	AAACTCAGCGCGAAGTCCACACCAC	Eurofins Genomics (Tokyo, Japan)
Genomic PCR primers		
Tyr-genome-fw	TATGTTTTGCTAAAGTGAGGTAAGAAAAGA	Eurofins Genomics (Tokyo, Japan)
Tyr-genome-rv	GTTATATAGGTCTTAGCCAAAACATGTGAT	Eurofins Genomics (Tokyo, Japan)
Hps4-genome-fw	GAGTTTGGTATTTTGCTGTCTTCCTGTTAT	Eurofins Genomics (Tokyo, Japan)
Hps4-genome-rv	AACCTGTCTCAAAACTTCAAAGATAAGTAA	Eurofins Genomics (Tokyo, Japan)
Rab27A-genome-fw	TGGCCAGTTTTAGGAATGTAATCTGTGGTT	Eurofins Genomics (Tokyo, Japan)
Rab27A-genome-rv	GTTTGACACTGAGTTGGCTCTTTTCTCTTT	Eurofins Genomics (Tokyo, Japan)
Rab32-genome-fw	GTCTTCCCGTTCTATTTGTTCACTT	Eurofins Genomics (Tokyo, Japan)
Rab32-genome-rv	CTAGATTTATCAGCGTACTCAGCCT	Eurofins Genomics (Tokyo, Japan)
Rab38-genome-fw	ATCTCTGTGAAAGGAGTAAAGCACT	Eurofins Genomics (Tokyo, Japan)
Rab38-genome-rv	ATTAAGCCAAACTTTCCAAGAGTGG	Eurofins Genomics (Tokyo, Japan)
RT-PCR primers		
Rab5A-Met	GGATCCATGGCTAATCGAGGAGCAAC	*J. Biol. Chem.* (2002) 277, 9212-9218
Rab5A-stop	TCAGTTACTACAACACTGGC	*J. Biol. Chem.* (2002) 277, 9212-9218
Rab5B-fw	CAGGCTGCAATCGTGGTCTA	Eurofins Genomics (Tokyo, Japan)
Rab5B-rv	TTCTGGGGTTCGCTCTTTGG	Eurofins Genomics (Tokyo, Japan)
Rab5C-Met	GGATCCATGGCGGGTCGAAGGAGGTGC	*Genes Cells* (2006) 11, 1023-1037
Rab5C-stop	TCAGTTGCTGCAGCACTGGC	*Genes Cells* (2006) 11, 1023-1037
Rab9A-Met	CGGATCCATGGCAGGAAAATCGTCTCT	*J. Biol. Chem.* (2002) 277, 9212-9218
Rab9A-stop	TCAACAGCAAGATGAGTTTG	*J. Biol. Chem.* (2002) 277, 9212-9218
Rab9B-Met	GGATCCATGAGTGGGAAATCCCTTCT	*Genes Cells* (2006) 11, 1023-1037
Rab9B-stop	CTAACAACAAGAAGAACTTG	*Genes Cells* (2006) 11, 1023-1037
Rab10-Met	CGGATCCATGGCGAAGAAGACGTACGA	*J. Biol. Chem.* (2002) 277, 9212-9218
Rab10-stop	TCAGCAGCACTTGCTCTTCC	*J. Biol. Chem.* (2002) 277, 9212-9218
Rab24-Met	CGGATCCATGAGCGGGCAGCGCGTGGA	*J. Biol. Chem.* (2003) 278, 15373-15380
Rab24-stop	TCAGTGATGACAACAGCTGT	*J. Biol. Chem.* (2003) 278, 15373-15380
Rab28-Met	CGGATCCATGTCAGACTCTGAGGAGGA	*J. Biol. Chem.* (2002) 277, 9212-9218

Rab28-stop	TCACTGCACTGCACACATGG	*J. Biol. Chem.* (2002) 277, 9212-9218
Rab30-Met	CGGATCCATGAGTATGGAAGATTATGA	*J. Biol. Chem.* (2003) 278, 15373-15380
Rab30-stop	TTAGTTGAAATTACAACAAG	*J. Biol. Chem.* (2003) 278, 15373-15380
Rab34-Met	CGGATCCATGAACATTCTGGCGCCCGT	*J. Biol. Chem.* (2002) 277, 12432-12436
Rab34-stop	TCAGGGACAACATGTGGCCT	*J. Biol. Chem.* (2002) 277, 12432-12436

Plasmids	RIKEN BioResource Research Center Cat#	Source
pEF-Tyr-FLAG	RDB14635	*J. Biochem.* (2017) 161, 323-326
pEF-T7-Hps4	RDB17366	*J. Biol. Chem.* (2019) 294, 6912-6922
pEGFP-C1-Rab27A	RDB17273	*J. Cell Sci.* (2006) 119, 2196-2203
pEF-T7-GST-Varp-ANKR1	RDB17371	*J. Biol. Chem.* (2016) 291, 1427-1440
pEF-T7-GST		*J. Biol. Chem.* (2016) 291, 1427-1440
pEGFP-C1-Varp-ANKR1	Constructed from pEF-T7-Varp-ANKR1 (see RDB17371)	*Mol. Biol. Cell* (200) 20, 2900-2908
pEGFP-C1-RUTBC1	RDB15018	*Genes Cells* (2009) 14, 41-52

Antibodies	Dilution	Source
Anti-Tyr rabbit polyclonal Ab	IB (0.054 μg/ml); IF (0.27 μg/ml)	*Traffic* (2011) 12, 627-643
Anti-Tyrp1 rabbit polyclonal Ab	IB (0.23 μg/ml)	*J. Invest. Dermatol.* (2013) 133, 2237-2246
Anti-Tyrp1 mouse monoclonal Ab	IF (1/1,000 dilution)	Santa Cruz Biotechnology (Dallas, TX) sc-58438
Anti-HPS4 rabbit polyclonal Ab	IB (1/200 dilution)	Proteintech (Rosemont, IL) 14627-1-AP
Anti-Rab27A rabbit polyclonal Ab	IB (4.8 μg/ml)	*Genes Cells* (2006) 11, 623-631
Anti-Rab32 rabbit polyclonal Ab	IB (5.8 μg/ml)	*Mol. Biol. Cell* (2009) 20, 2900-2908
Anti-Rab38 mouse monoclonal Ab	IB (1/1,000 dilution)	Abnova (Tipei, Taiwan) H00023682-M02
Anti-FLAG tag (M2)-HRP monoclonal Ab	IB (1/5,000 dilution)	Sigma-Aldrich (St. Louis, MA) A8592
Anti-FLAG tag (M2) monoclonal Ab	IF (1/1,000 dilution)	Sigma-Aldrich (St. Louis, MA) F3165
Ant-GFP polyclonal Ab	IB (1/1,000 dilution)	MBL (Nagoya, Japan) 598
Anti-GST mouse monoclonal Ab	IB (0.2 μg/ml)	Santa Cruz Biotechnology (Dallas, TX) sc-138
Anti-Rab10 (D36C4) mouse monoclonal Ab	IB (1/100 dilution)	Cell Signaling Technology (Danvers, MA) 8127
Anti-Rab24 rabbit polyclonal Ab	IB (1.08 μg/ml)	*J. Cell Biol.* (2016) 213, 355-369
Anti-Rab34 rabbit polyclonal Ab	IB (10.6 μg/ml)	*J. Cell Biol.* (2016) 213, 355-369
Goat Anti-Mouse IgG(H+L), Human ads-HRP	IB (1/10,000 dilution)	SouthernBiotech (Birmingham, AL) 1031-05
HRP-anti-rabbit IgG	IB (1/10,000 dilution)	GE Healthcare (Chicago, IL) NA934-1ML
Alexa Fluor plus 488-conjugated anti-mouse IgG	IF (1/1,000 dilution)	Thermo Fisher Scientific (Waltham, MA) A32723
Alexa Fluor plus 488-conjugated anti-rabbit IgG	IF (1/1,000 dilution)	Thermo Fisher Scientific (Waltham, MA) A32731
Alexa Fluor plus 555-conjugated anti-mouse IgG	IF (1/1,000 dilution)	Thermo Fisher Scientific (Waltham, MA) A-32727
Alexa Fluor plus 555-conjugated anti-rabbit IgG	IF (1/1,000 dilution)	Thermo Fisher Scientific (Waltham, MA) A32732

Ab, antibody; fw, forward; IB, immunoblot; IF, immunofluorescence; rv, reverse.
